# Reorganizing the Intrinsic Functional Architecture of the Human Primary Motor Cortex during Rest with Non-Invasive Cortical Stimulation

**DOI:** 10.1371/journal.pone.0030971

**Published:** 2012-01-27

**Authors:** Rafael Polanía, Walter Paulus, Michael A. Nitsche

**Affiliations:** Department of Clinical Neurophysiology, Georg-August University of Göttingen, Göttingen, Germany; City of Hope National Medical Center and Beckman Research Institute, United States of America

## Abstract

The primary motor cortex (M1) is the main effector structure implicated in the generation of voluntary movements and is directly involved in motor learning. The intrinsic horizontal neuronal connections of M1 exhibit short-term and long-term plasticity, which is a strong substrate for learning-related map reorganization. Transcranial direct current stimulation (tDCS) applied for few minutes over M1 has been shown to induce relatively long-lasting plastic alterations and to modulate motor performance. Here we test the hypothesis that the relatively long-lasting synaptic modification induced by tDCS over M1 results in the alteration of associations among populations of M1 neurons which may be reflected in changes of its functional architecture. fMRI resting-state datasets were acquired immediately before and after 10 minutes of tDCS during rest, with the anode/cathode placed over the left M1. For each functional dataset, grey-matter voxels belonging to Brodmann area 4 (BA4) were labelled and afterwards BA4 voxel-based synchronization matrices were calculated and thresholded to construct undirected graphs. Nodal network parameters which characterize the architecture of functional networks (connectivity degree, clustering coefficient and characteristic path-length) were computed, transformed to volume maps and compared before and after stimulation. At the dorsolateral-BA4 region cathodal tDCS boosted local connectedness, while anodal-tDCS enhanced long distance functional communication within M1. Additionally, the more efficient the functional architecture of M1 was at baseline, the more efficient the tDCS-induced functional modulations were. In summary, we show here that it is possible to non-invasively reorganize the intrinsic functional architecture of M1, and to image such alterations.

## Introduction

Motricity is the fundamental mechanism that mammals use to interact with the environment. The control of such motor actions is carried out by the primary motor cortex (M1), whose output functions are somatotopically ordered in a medial-to-lateral representational map with three major subregions: leg, arm and face [1]. More recent studies accept the subdivision of these three major M1 regions, but reject the idea of a precise topography with discrete representations, and instead show that M1 is best described as a broadly distributed network involving large populations of neurons between and within subregions [2], . Following this concept, the intrinsic organization of M1 was shown to have distributed and overlapping representations which are suggestive of intrinsic substrates for learning of motor skills accompanied by functional reorganization [5], [6]. This dynamic functional architecture of M1 appears to be related with long-lasting changes of the efficacy of intrinsic horizontal connections, whose foundation is thought to be long-term potentiation (LTP) and long-term depression (LTD) [7], [8], [9].

Transcranial direct current stimulation (tDCS) is a non-invasive brain stimulation tool suited to alter cortical excitability and activity via application of direct currents. Anodal tDCS over the motor cortex during rest has been shown to increase and cathodal tDCS to decrease excitability of this area [10], [11]. Interestingly, the after-effects of tDCS are NMDA receptor-dependent [12], [13], [14], thus sharing some similarities with LTP, and LTD, which resemble well-known neuroplastic alterations thought to underlie cognitive processes like learning and memory formation [15]. In line with these studies in humans, anodal tDCS over M1 was shown to promote synaptic plasticity in rat brain slices, producing synaptic LTP [16]. In accordance, anodal tDCS improves motor learning and non-dominant hand function in healthy subjects [17], [18], [19], as well as facilitate performance of motor skills in stroke patients with respective deficits [20], [21], [22].

In prevoious imaging studies the impact of tDCS over M1 have been studied at the large-scale level (i.e. studying whole brain interactions), where it has been reported that excitatory anodal tDCS is capable of modulating motor-task related cortico-cortical [23], [24], [25] and cortico-subcortical [26] functional circuits. However it has been not yet stablished how tDCS-induced neuroplasticity over M1 intra-regionally reorganizes its functional architecture.

In the present study we hypothesized that the relatively long-lasting synaptic modification induced by tDCS over M1 results in the alteration of associations among populations of M1 neurons which may be reflected in a change of its functional architecture. Such a tDCS-generated alteration of intrinsic connectivity might help to explain the previously reported impact of tDCS on motor learning. To test this hypothesis, we explored tDCS-related changes of functional M1 connectivity by aid of spontaneous BOLD fMRI activity. M1 functional networks were characterized using graph theory at voxel level resolution. Graph parameters that provide useful information regarding the functional architecture (e.g. connectivity degree, clustering coefficient and characteristic path-length [27], [28], [29], [30], [31], [32]) of M1 were computed and compared at the global and nodal level before and after anodal, cathodal or sham stimulation sessions.

## Methods

### Subjects

14 healthy volunteers (8 women; mean age 26±4 years; age range 21–40 years) were included in the study. Subjects were informed about all aspects of the experiments and all gave informed consent before participation. None of the subjects suffered from any neurological or psychological disorder, had metallic implants/implanted electric devices, or took any medication regularly, or in the 2 weeks before participation in any of the experiments. All subjects were right-handed, according to the Edinburgh handedness inventory [33]. The experiments conform to the Declaration of Helsinki, and the experimental protocol was approved by the Ethics Committee of the University of Göttingen.

### tDCS

Direct current was provided via a pair of square rubber electrodes (7×5 cm) compatible to be used in MR-scanner environment, which were connected to a specially developed battery-driven stimulator outside the magnet room (NeuroConn GmbH, Ilmenau, Germany). Further technical details regarding the characteristics of the stimulator can be found elsewhere [23], [25]. In order to properly position the electrodes over the M1 of the subjects' head, the representational field of the right hand was determined using suprathreshold TMS (optimal M1 representation of the right first dorsal interosseous muscle (FDI) by single pulse TMS). Before subjects entered the MR scanner, for anodal stimulation over M1, the anodal tDCS electrode was placed over the respective left M1 hand area and the cathode above the contralateral right orbit using conventional electrode cream. For cathodal stimulation over M1, the current flux was reversed. tDCS was applied for 10 minutes at 1 mA current intensity inside the MRI scanner. For sham stimulation sessions, the current was applied for 30 seconds at the beginning of the stimulation and then turned off (20 seconds linear down-ramping until 0 mA was reached). Using this placebo stimulation technique subjects are not able to distinguish between real and sham stimulation [34]. The rationale to target tDCS over the dominant hemisphere is that functional connectivity of this hemisphere is expected to be larger than that of the non-dominant one [35]. Moreover, this electrode montage – anode over the M1 and cathode over the contralateral frontopolar cortex – has been shown to be the optimal montage to enhance excitability of the motor cortex [36].

### fMRI

fMRI was conducted in a 3 Tesla scanner (Magnetom TIM Trio, Siemens Healthcare, Erlangen, Germany) using a standard eight-channel phased array head coil. Subjects were placed supine inside the magnet bore and wore headphones and additional ear plugs for noise protection. Initially, anatomic images based on a T1-weighted 3D turbo fast low angle shot (FLASH) MRI sequence at 1 mm^3^ isotropic resolution were recorded (repetition time (TR) = 2250 ms, inversion time: 900 ms, echo time (TE) = 3.26 ms, flip angle: 9°). For BOLD fMRI, a multislice T2*-sensitive gradient-echo echo-planar imaging (EPI) sequence (TR = 1800 ms, TE = 30 ms, flip angle 70°) at 3×3 mm^2^ resolution was used. Twenty nine consecutive sections at 3 mm thickness, angulated in an axial-to-coronal orientation, covering the whole brain, were acquired. 175 contiguous EPI volumes were acquired for each fMRI data set i.e. ∼6 minutes resting fMRI. After the initial T1 dataset acquisition, two resting-state fMRI datasets were acquired immediately before and after the application of tDCS inside the MRI scanner. The tDCS electrodes were disconnected from the stimulator during fMRI acquisition. No distortion was seen in the images as reported previously [25]. fMRI images were acquired before and after, but not during tDCS application. Subjects were asked to relax, keep their eyes closed and “not to think about anything in particular”. Each subject underwent three sessions: anodal, cathodal and sham stimulation; the order of sessions was interindividually randomized and the single sessions were separated at least 8 days from each other. Subjects were blinded for the stimulation conditions in order to control for possible placebo effects. Thus, altogether 84 resting state fMRI data sets were acquired i.e. N = 14 subjects * tDCS session (anodal, cathodal, sham) * time (before and after tDCS)

### MRI and fMRI pre-processing

**Figure 1:**
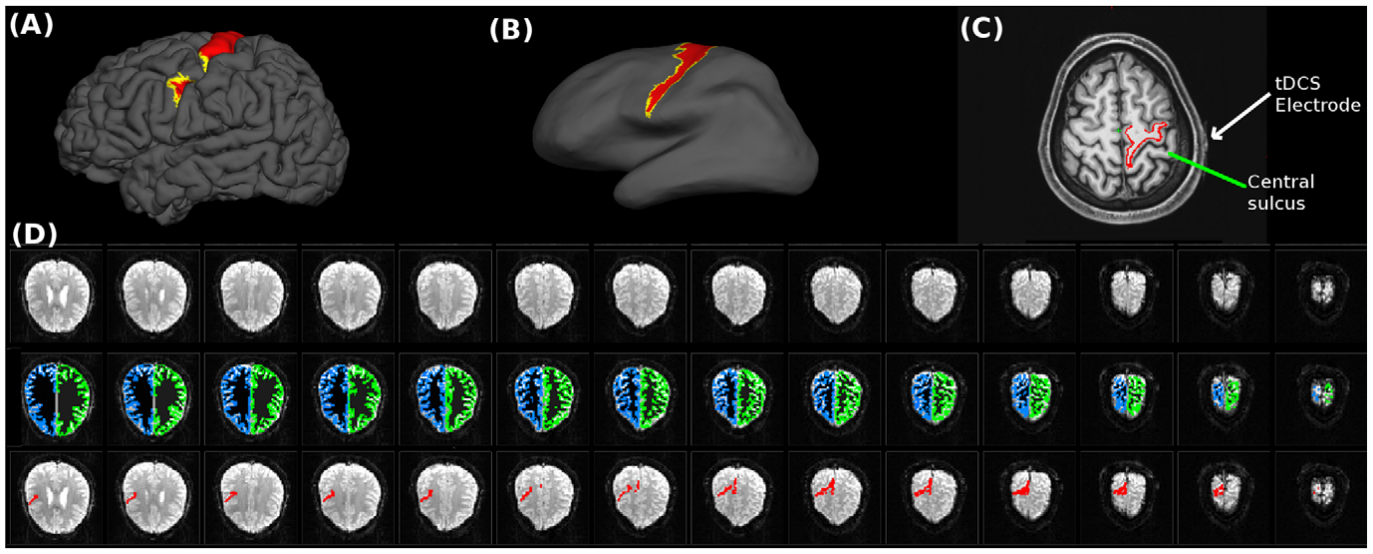
BA4 labelling and registration to the functional space. An example of the quality of the cortical segmentation and labelling of BA4 is depicted, which was performed with Freesurfer in one of the participants. Panels **A** and **B** show the BA4 label over the pial and inflated brain surface respectively. Panel **C** shows the left BA4 label registered in the original T1 image. The red line shows the portion of the cortical segmentation performed by Freesurfer that corresponds to the left BA4 label (in this image left is right). Notice the tDCS electrode over the scalp of the subject is positioned over the central sulcus. Panel **D** shows the registration of the cortical segmentation and BA4 label in the native functional space of the same subject (first row: first 14 images of the native functional space. Second row: cortical segmentation registered from Freesurfer to the native functional space was overlapped. Third row: BA4 label (red) registered from Freesurfer space to the native functional space). For visualization purposes, we show the approximate location of the tDCS electrode over left BA4 in figure S1.

The first step was to perform cortical segmentation and labelling of the left BA4. In order to take into account variations in cortical folding across subjects, cortical segmentation of the T1 sequence was carried out in a standard spherical surface space, performed with the Freesurfer software package (http://surfer.nmr.mgh.harvard.edu/). The cortical segmentation was visually inspected for each subject by overlapping the grey matter (GM) - white mater (WM) boundary over the T1 image. No large misclassification of white matter or cerebral spinal fluid (CSF) voxels as grey matter voxels was found in any of the individual T1 images. After segmentation completion, Freesurfer generates surface labels for some cytoarchitectonic brain regions including BA4a and BA4p. The generated surface labels left-BA4a and left-BA4p were merged into a single label BA4. Afterwards, the surface label BA4 was transformed back to the original T1 space, visually inspected and manually corrected, if necessary (figure 1). For visualization purposes, we show the approximate location of the tDCS electrode over left BA4 in figure S1. The next step was pre-processing of the functional datasets.

All functional pre-processing steps were carried out with the FSL software package (http://www.fmrib.ox.ac.uk/fsl/). The first two volumes of each fMRI dataset were discarded to allow for magnetization equilibrium. Motion correction was applied using MCFLIRT and slice-timing correction using Fourier-space time-series phase-shifting [37]. Because the graph theoretical analysis was performed at the voxel level, no spatial filtering or spatial normalization was performed in order to avoid introduction of artificial correlations between neighbouring voxels. Additionally, to control for physiological processes and motion-related artefacts in the functional connectivity analysis [38], we regressed the following nine signals from each subject's 4-D datasets: the six motion parameters, the nuisance parameters from the white matter WM, CSF and the global signal. The regression of CSF and WM removes fluctuations unlikely to be involved in specific regional correlations. Additionally, the whole brain signal is thought to reflect a combination of physiological processes (such as cardiac and respiratory fluctuations) and scanner drift [39], [40]. Correction for time series autocorrelation (prewhitening) was performed. The six motion parameters were generated by MCFLIRT. The global signal parameter was generated by averaging across all voxels within the brain. We assured that for each subject the root mean square (rms) of the movement parameters did not exceed 1 mm or 1° in any of the cardinal directions or rotational axes.

To generate the WM and CSF nuisance parameters we first segmented each subject's T1 weighted high-resolution image using the FAST segmentation program in FSL. The resulting segmented WM and CSF images were thresholded to ensure 90% tissue type probability. The thresholded masks were applied to each subject's time series and the mean time series was calculated by averaging across all voxels within the mask. This nuisance signal regression procedure produced prewhitened, 4D residual datasets for each subject.

Afterwards, the surface label BA4 was re-sliced and co-registered to the 3×3×3 mm native resolution of the resting fMRI time-series of each resting fMRI dataset using an FSL-Freesurfer interface registration program that uses a complex convex hull algorithm to optimize the co-registration between the segmented T1 images to the FSL native functional space (http://surfer.nmr.mgh.harvard.edu/). The co-registered BA4 label was masked in order to identify the voxels in the native functional space that belong to BA4. Finally, the functional time-series belonging to the BA4 mask were band-pass filtered with a zero-lag band-pass filter to select the low resting state frequencies of interest (0.01–0.09 Hz). After pre-processing, the fMRI time-series belonging to the BA4 mask were analysed using graph theory.

### Graph theory

**Figure 2:**
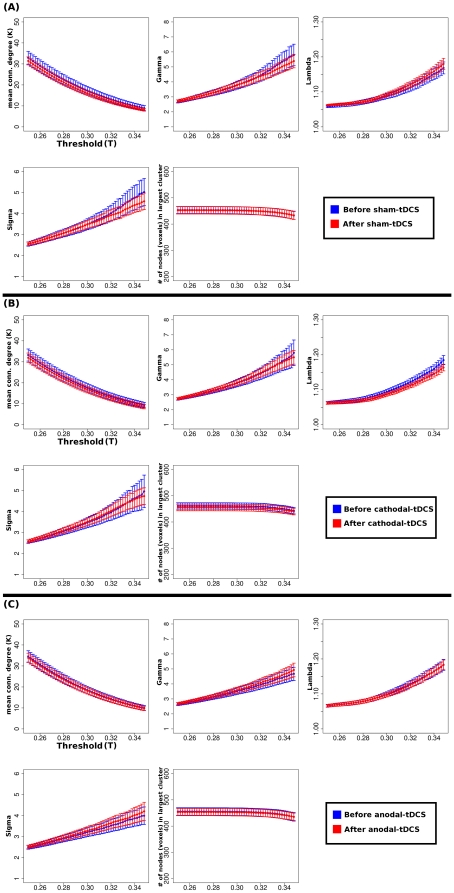
Global network metrics. Shown are the results of the global network parameters that were calculated in the present study (mean connectivity degree (*K*) and the small-world parameters gamma, lambda and sigma [32]) calculated at each threshold T (0.25–0.35 in increasing steps of 0.002) before and after each Sham (A), Cathodal (B) and Anodal (C) tDCS. The second row - second column of each panel show that the approximate number of nodes (M1 voxels) of each undirected graph was ∼470. As expected, the mean connectivity degree monotonically decreases as *T* increases. M1 has salient small-world properties i.e. *lambda≈1*, *gamma≫1*, thus *sigma≫1* [32], [41]. No significant differences were observed for any of the network metrics before and after each of the tDCS sessions (P>0.05 paired two-tailed t-tests). Error bars represent the s.e.m.

Zero-lag temporal correlations between all pair-wise combinations of the functional time-series belonging to the BA4 mask were computed, resulting in an *N×N* synchronization matrix *M* for each functional data set – before and after tDCS. *N* was about 470 across the group of subjects (Figure 2, second row - second column of each panel). Then, for each *M* a connectivity graph *G* was formed consisting of *N* nodes and a set of undirected edges *E* (functional connectivity) by applying a correlation threshold *T* to *M*:
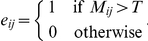
Hence, if the zero-lag correlation value between a pair of grey matter voxels 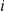 and 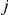 is greater than the given value 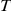, an edge is said to exist. It is important to notice that it was convenient to build the synchronization matrices *M* in the native functional space rather than in the normal space in order to avoid the introduction of artificial correlations due to spatial normalization (for more details please see [25]). Each *M* was thresholded starting at *T = 0.1* in steps of 0.002 until the largest connected cluster included more than 95% of all nodes in *G*, thus obtaining a *T_max_* for each data set, and 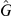 formed by the largest cluster. For each 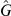, we computed graph parameters that provide useful information regarding the functional architecture of the network (BA4). In the present study we initially computed the following global network properties: the mean connectivity degree *K*, which is the average of edges (functional connections) per node (BA4 voxel); the clustering-coefficient *C*, which provides information about the efficacy of the local connectedness of the network; and the characteristic path-length *L*, which provides information about the efficacy of global network communication [27], [28], [31]. Small-world properties were calculated by comparing the absolute cluster coefficient and the absolute path lengths between the experimentally altered and random networks. That is *gamma = C/C_rand_*>1 and *lambda* = *L/L_rand_*≈1, finally obtaining the small-worldness coefficient ratio *sigma* = *gamma/lambda* [32]. The theoretical values that can be used for random clustering and path length coefficients are: *C_rand_ = K/N* and *L_rand_ = ln(N)/ln(K)* [41]. However, these theoretical networks have Gaussian distributions and might not provide accurate values when we compare them with our experimental networks which might have totally different distributions. In order to control for that, we built random networks which preserved the degree of distributions of our experimental networks [32]. For each threshold *T*, the values for *C_rand_* and *L_rand_* were calculated as the average of the computed graph characteristics of 1000 random graphs.

Next, we used the minimum *T_max_* obtained from all data sets and all subjects to construct undirected connectivity graphs 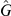 from the largest connected cluster for all subjects (at least 95% from all of the nodes should belong to the largest cluster, see above) and subsequently built volume maps using the graph theory metrics. From 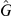, we built volume maps in the native functional space within the BA4 mask using: (1) the nodal connectivity degree *K_i_* (*K* map), where *K_i_* is the number of direct neighbours of *i*; (2) the nodal clustering-coefficient *C_i_* (*C* map), where *C_i_* is defined as:
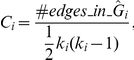
Where 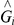 is the subgraph formed by the node *i* and its direct neighbours and *k_i_* is the number of edges of voxel *i*; and (3) the nodal characteristic path length *L_i_* (*L* map), where let *d(i,j)* be the minimum functional distance between voxel *i* and *j* i.e. the minimal number of edges needed to travel from voxel *i* to voxel *j*. The nodal minimum path length *L_i_* is defined as:
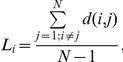
which provides information regarding the functional connectivity distance between voxel *i* with all the other voxels in BA4 i.e. how well the voxel *i* is globally-functionally linked with all the other grey matter voxels in BA4 (for further information regarding graph theoretical parameters see: [28], [32], [42]). The individual *K* volume maps were then scaled from 0 to 1, by dividing the *K* values by the maximum value of the individual map to normalize the values over the group of subjects. The *C* volume maps were normalized by applying *C_i_/C_rand_*. In contrast, the *L* volume maps were normalized by applying *L_rand_/Li* (the reason for this was that the lower *L_i_* is, the better the node *i* is connected to the rest of the network; thus for visualization it is intuitively more convenient if higher connectivity according to the parameter *L* is indicated by larger values in the *L* volume maps). Afterwards, multi-subject statistics for the *K*, *C* and *L* maps were carried out in the standard spherical surface space – the Freesurfer surface space – instead of the normal 3D space, thus eliminating a large source of inter-subject variability (variations in cortical folding across subjects). To this end, the normalized *K*, *C* and *L* maps were transformed back from the native functional space to the Freesurfer space by using the information of the transformation matrix in the initial registration (see fMRI pre-processing section). In the surface space the individual *K*, *C* and *L* maps were smoothed with a 6 mm FWHM smoothing kernel and transformed to the mean surface of the brains of the subjects included in the present study.

### Statistical analysis

Initially, the global network parameters *K*, *C* and *L* were compared before and after each stimulation condition using paired t-tests for all thresholds *T* (starting at *T* = 0.1 in increasing steps of 0.002). Afterwards, statistical comparisons for *K*, *C* and *L* surface maps were performed in the surface space by initially carrying out a repeated measures ANOVA (for both factors Stimulation and time), and evaluating the interaction effects (stimulation*time). Only if an effect of interaction was found, we conducted post-hoc paired t-tests. ANOVAs and paired t-test maps were thresholded at uncorrected p<0.05 and the resulting clusters were then p<0.05 Monte-Carlo corrected (5000 random permutations). All computations performed in this study were done off-line by in-house software written by one of the authors (RP) fully developed under: FSL, Freesurfer, R (http://www.r-project.org/) and C++ compiled using gcc version 4.3.2 under Linux i386.

## Results

The first step is to analyze the global characteristics of the graphs representing the M1 networks. The approximate number of grey matter voxels belonging to the left M1 region was ∼470. Before and after tDCS, we found that the mean connectivity degree monotonically decreases as the threshold is incremented, which is typical for brain networks [41]. Additionally, the ratio of the clustering coefficient in the M1 experimental networks compared to random networks is *gamma≫1*, meaning that local M1 networks efficiently communicate at the local level. On the other hand, when the characteristic path lengths where compared, the ratio between real and random networks (*lambda*) showed to be approximately 1, suggesting that M1 has an efficient segregated functional connectivity. With these results, it is not surprising that the ratio *sigma = gamma/lambda* to be much larger than 1, suggesting that M1 has small-world properties (Figure 2). Afterwards, all studied global network parameters (mean connectivity degree *K*, and the small-world parameters *gamma*, *lambda* and *sigma*) were compared by performing paired t-tests. Here no significant differences were found (P>0.05) at all the studied thresholds *T* (Figure 2). For visualization purposes we show in figure S2 the left BA4 connectivity matrices for one of the subjects in all of the six resting state conditions (time*stimulation).

**Figure 3:**
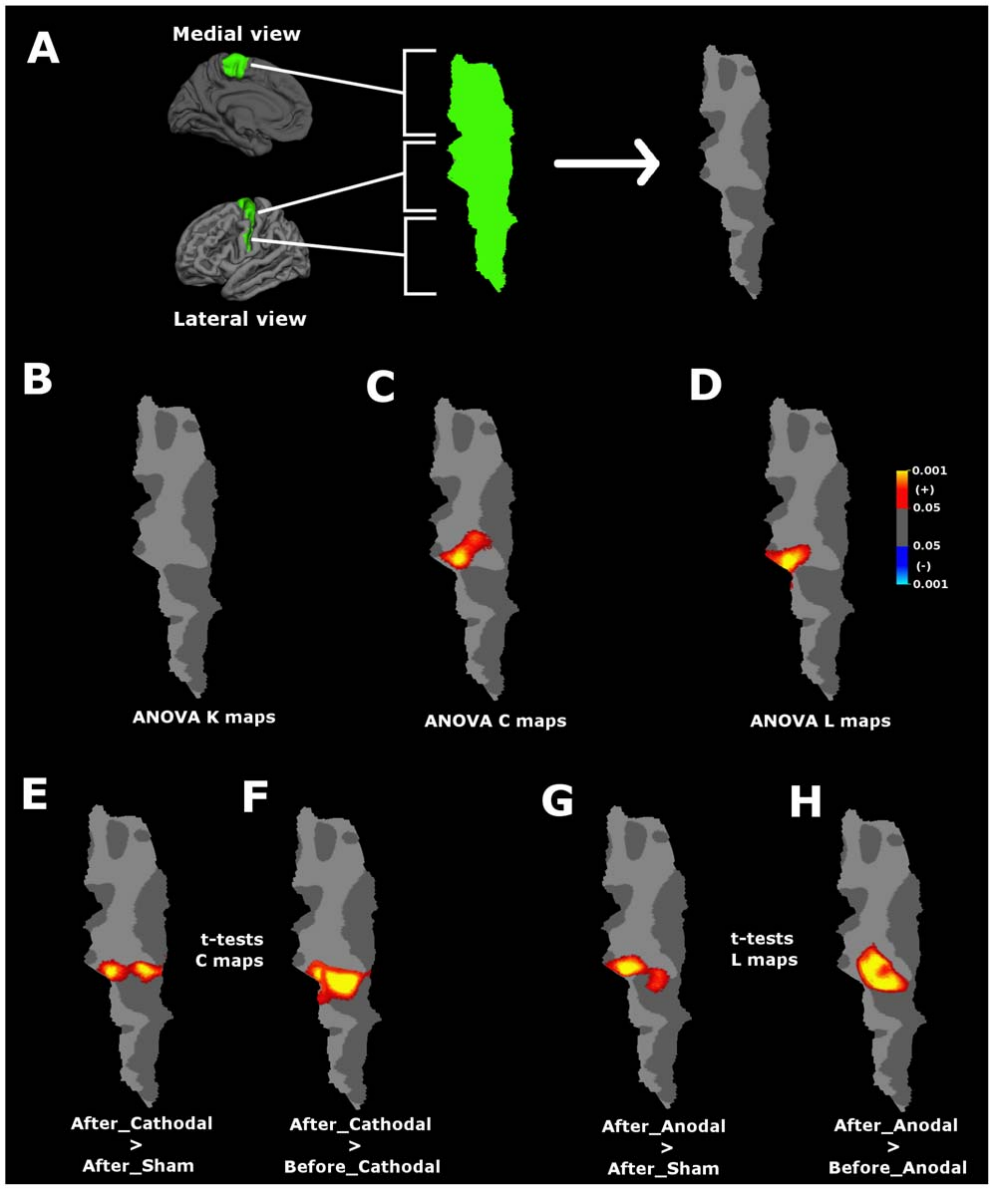
Graph parameter statistics at the BA4 cortical surface. (A) Shown is the flattening of the left BA4 (green labelled region) obtained from the left hemisphere surface average subject, which was used to project the statistical maps. Panels B to D show the ANOVA for the interaction effects (time*stimulation) Montecarlo cluster corrected at p<0.05 for the nodal connectivity degree maps (B), clustering coefficient maps (C) and the characteristic path length maps (D). Panels E to H show post hoc paired t-tests for the following contrasts: (E) After_Cathodal – After_Sham in the clustering coefficient maps; (F) After_Anodal – Before_Anodal in the clustering coefficient maps; (G) After_Anodal – After Sham in the characteristic path length maps; (H) After_Anodal – Before_Anodal in the characteristic path length maps. Notice that the *L* maps were *L_rand_/Li* normalized, which means that the values of *L* in the significant cluster are lower after stimulation (see methods section).

The second step was to perform an analysis at the nodal level. Therefore, a threshold common for all subjects was selected as described in detail in the methods section. The maximum *T* that included at least 95% of the nodes in the largest cluster across all of the subjects was *T = 0.352*. Therefore, we used this threshold to build the undirected graphs and subsequently generate volume/surface maps with each network metric (*K*, *C* and *L*) for all of the resting state data-sets before and after tDCS. For visualization purposes, the undirected graph representation for one of the correlation matrices thresholded at *T = 0.352* is shown in Figures S3 and S4. The ANOVAs calculated in the surface space revealed a significant interaction effect (stimulation*time) for the clustering coefficient (Talairach x = −40,y = −9,z = 57, peak F-value = 5.1, p<0.005; cluster size 439 mm^2^; figure 3C) and the characteristic path length (Talairach x = −39,y = −11,z = 55, peak F-value = 5.9, p<0.005; cluster size 392 mm^2^; figure 3D). These significant clusters are located in the dorsolateral BA4 and approximately belonging to the arm/hand area according to an fMRI M1 mapping carried out by Meier et al. [43]. With regard to the connectivity degree, we did not find any interaction effect (figure 3A). After Monte-Carlo cluster correction (P<0.05) to the paired t-tests statistical clustering coefficient maps after-before of each stimulation condition, we identified one positive cluster in the cathodal stimulation condition (Talairach x = −39,y = −11,z = 56, peak t-value = 5.2, P<0.001; cluster size 499 mm^2^; figure 3F). The After – Before contrasts applied to anodal and sham conditions did not show any significant cluster. A re-test paired t-test analysis to the contrast After_Cathodal_ – After_Sham_ also revealed a positive cluster located at approximately the same location of the ANOVA test (Talairach x = −39,y = −11,z = 57, peak t-value = 5.2, p<0.005; cluster size 468 mm^2^; figure 3E). T-tests applied to the baselines between conditions did not reveal any significant cluster. The same analysis was repeated for the paired t-tests on the characteristic path length maps. The After_Anodal_ – Before_Anodal_ contrast revealed a positive cluster (Talairach x = −37,y = −13,z = 53, peak t-value = 4.7, p<0.005; cluster size 520 mm^2^; figure 3H). The After – Before contrasts applied to cathodal and sham conditions did not show any significant cluster. A retest paired t-test analysis to the contrast After_Anodal_ – After_Sham_ also revealed a positive cluster located at approximately the same location of the ANOVA test (Talairach x = −40,y = −9,z = 55, peak t-value = 4.9, p<0.005; cluster size 451 mm^2^; figure 3G). T-tests applied to the baselines between conditions did not reveal any significant cluster.

In an exploratory analysis, we investigated whether tDCS applied over the left M1 may have resulted in functional connectivity changes of the contralateral M1. Thus, we repeated the whole analysis (MRI-fMRI pre-processing and graph theory) using the right BA4. However we did not find any significant alterations of functional connectivity with regard to this area (no significant cluster showed up using ANOVA and evaluating the interaction group(anodal, cathodal, sham) * time(pre- and post-tDCS) for any of the nodal network parameters used in the present study (nodal connectivity degree, characteristic path length and clustering coefficient)). This lack of tDCS-induced functional reorganization in the contralateral hemisphere might correlate with a study of Lang and colleagues [44], where exactly the same electrode size (5×7 cm), stimulation intensity (1 mA) and duration (10 min) were applied; however the investigators failed to find changes of cortical excitability in the opposite hemisphere (also the right M1) – the authors only observed some weak effect in interhemispheric inhibition. One possible explanation for this is that interhemispheric connections have higher thresholds than local cortico-cortical and cortico-spinal connections [45], hence a lack of contralateral excitability. Additionally, it might be also possible that the intensity of 1 mA is too weak to modulate transcallosal activity. These are important points that should be examined in future studies by combining functional reorganization evaluated with graph theory accompanied by electrophysiological measures.

**Figure 4:**
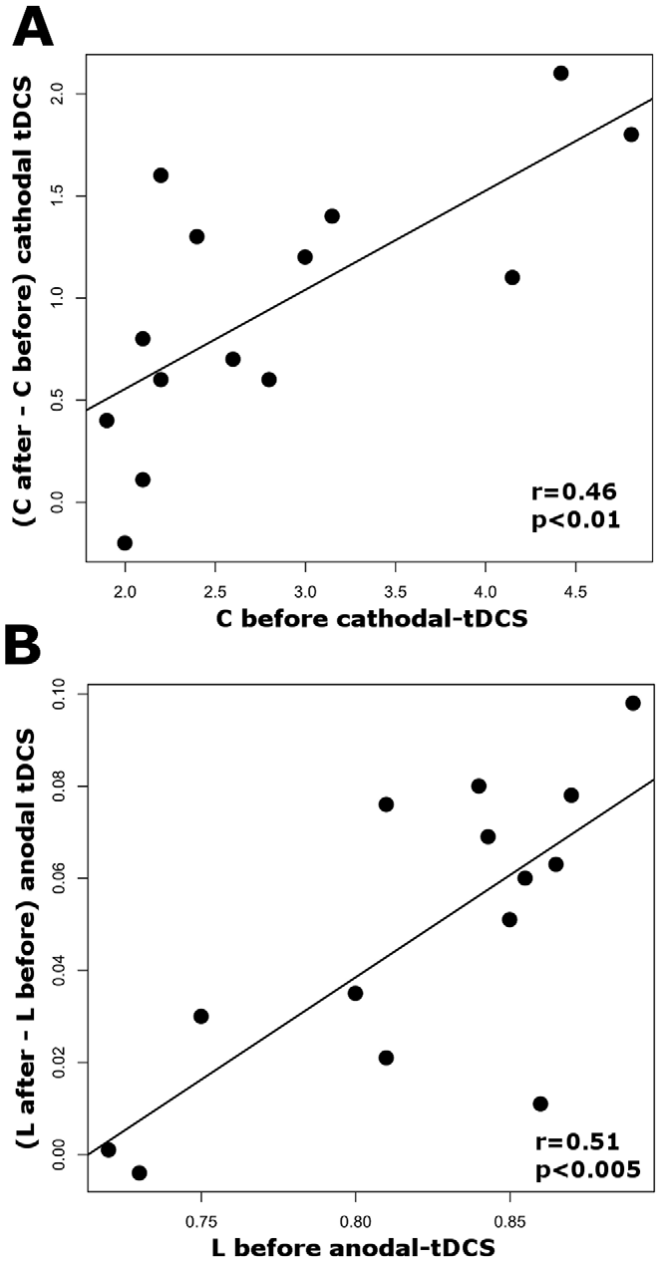
Dependency of the tDCS-induced effects on baseline functional architecture. Panel A shows that the effect of nodal clustering coefficient (*C*) increase found in the cluster of Figure 3F strongly correlated with baseline *C* (P = 0.0051; R^2^ = 0.46). Panel B shows that the positive decrease found in the characteristic path length (*L*) maps that was found after anodal tDCS (Figure 3H) also has a positive correlation with the baseline *L* (P = 0.002; R^2^ = 0.51).

In a *post-hoc* analysis, we investigated whether the tDCS-induced functional connectivity alterations observed in the *C* and *L* maps depended on the initial functional network metric of each subject. The average of the effect within the cluster (after-before within tDCS) was computed for each subject and then these values were linearly regressed against the baseline value of its respective network metric, e.g. the after-before mean values of the significant cluster found in the *C* Maps in the cathodal tDCS condition were regressed against the before cathodal *C* maps. The effect of a clustering coefficient increase following cathodal tDCS strongly correlated with the baseline *C* (P = 0.0051; R^2^ = 0.46) (Figure 4A). The negative increase in the characteristic path length that was found after anodal tDCS also correlated positively with the baseline metric (P = 0.002; R^2^ = 0.51) (Figure 4B). Regressions applied to the same clusters in the sham condition did not result in any significant correlation.

**Figure 5:**
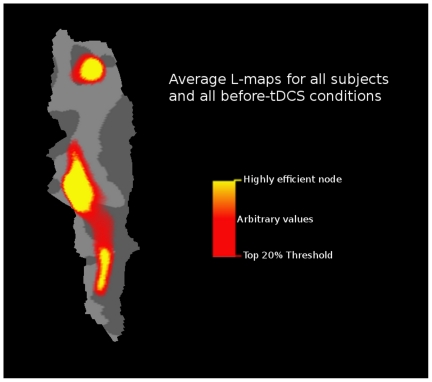
Highly efficient nodes within M1. The average *L* maps for all subjects and all before-tDCS fMRI scans were averaged. Nodes that showed the highest *L_rand_/Li* values (i.e. nodes that communicate more efficiently within M1) were mapped over the flattened BA4. As an exploratory threshold we used the 15% of the voxels that showed the highest *L_rand_/Li* values.

In a second *post hoc* analysis we investigated the reason to have such localized effect of tDCS in the dorsolateral BA4 region (Figures 3C–H). We hypothesized that the reason for the arm/hand region to be significantly altered by tDCS during rest is that this is the M1 region with the most efficient dynamic architecture. We mapped the nodes that communicate more efficiently independently from stimulation (i.e. before tDCS intervention) within the M1 network. To this end, the average *L* maps for all subjects and all before-tDCS fMRI scans were averaged. Nodes with the highest *L_rand_/Li* values (i.e. nodes that communicate more efficiently within M1) were mapped over the flattened BA4. As an exploratory threshold we used the 15% of the voxels that showed the highest *L_rand_/Li* values. The largest hub was located at the centre of the flattened BA4 area, which approximately represents the arm/hand area according to an fMRI M1 mapping carried out by Meier et al. [43] (peak value at Talairach x = −36,y = −15,z = 55). Two additional smaller hubs were identified: one belonging to approximately the leg area (cluster at the top of the flattened BA4 (medial BA4); Talairach x = −36,y = −15,z = 55); and a second cluster belonging approximately to the face/tongue area (cluster at the bottom-tight of the flattened BA4 (most lateral BA4); Talairach x = −36,y = −15,z = 55) according to an fMRI M1 mapping carried out by Meier et al. [43] (Figure 5).

## Discussion

Here we have shown via fMRI and graph theoretical functional connectivity analysis that: (a) M1 is functionally organised in a highly efficient and distributed way; (b) at the arm/hand M1 region cathodal tDCS boosts local connectedness, while anodal tDCS boosts long distance functional connections within M1; and (c) the more efficient the functional architecture of M1 at the arm/hand region is at baseline, the more efficient the tDCS-induced functional modulations are. In the following, we will discuss each of these points in more detail.

We tested the hypothesis that tDCS-induced neuroplasticity over M1 results in an alteration of associations among populations of M1 neurons, reflected in a change of its functional architecture. However, before it was important to show that the functional connections of M1 are organized in distributed and efficient way, rather than having a lattice organization. For stimulation-independent functional connectivity of M1 during rest, the results show that M1 has salient small-world properties, i.e. *lambda≈1, gamma≫1*, thus *sigma≫1* [32], [41], indicative for highly efficient integration of both, localized and segregated information processing, in M1. If M1 functional organization would reflect a precise topography with discrete representations, we would have expected reduced segregated connectivity (i.e. lambda≫1 [32]), which is clearly not the case. This finding is important, because this kind of functional architecture should be relatively flexible for modification and map reorganization. In accordance, it has been shown that the control and learning of simple and complex voluntary movements emerge from M1 distributed networks rather than discrete representations [46].

Hence, the second step was to explore if tDCS-induced neuroplasticity of M1 is reflected in a modulation its functional architecture. At first instance, Figure 2 shows a lack of global alterations of functional connectivity of M1 induced by tDCS, i.e. the global M1 network parameters remained constant (for all studied thresholds no significant changes in the global mean connectivity degree *K*, as well as the small-world properties were obtained (Figure 2)). This means that the application of relatively weak constant currents (1 mA) over the scalp of healthy humans for few minutes still preserves the global functional structure of M1. However, when we compared the surface maps that contained the information of the nodal network metrics, we found significant changes at local clusters within M1 following tDCS. Cathodal stimulation induced an increase in the clustering coefficient at the dorsolateral BA4 – approximately the M1 arm/hand area. It should be noticed that this effect was not accompanied by a significant modulation in the connectivity degree (i.e. the number of functional connections did not significantly increased or decreased). This means that the strength of the functional connections at the local level was significantly increased by cathodal tDCS. This is an interesting finding considering that the primary mechanisms of the excitability shifts induced by tDCS are subthreshold neuronal membrane depolarization by anodal stimulation and membrane hyperpolarization by cathodal stimulation, and similar consecutive alterations of spontaneous cortical activity as shown directly in animal studies [47], [48], but also suggested for tDCS of the human motor cortex [13]. A likely explanation for this result might be that the local decrease of spontaneous activity induced by cathodal tDCS increased the signal to noise ratio (by inducing neuronal hyperpolarization) and consequently increased synchronization at the local level. This hypothesis might be supported by previous studies where cathodal tDCS targeted at other brain regions is suggested to increase signal to noise ratio e.g. the motion processing areas in the visual cortex [49]. Following this concept, we would expect that anodal tDCS might have induced a decrease in local synchronization at M1 level due to a reduction of the signal to noise ratio. Although for anodal tDCS we did not find any region where the clustering coefficient or connectivity degree significantly increased or decreased, the characteristic path length significantly decreased in a cluster centred – again – at approximately the arm/hand area of M1. This means that the nodes belonging to that cluster communicate more efficiently with the rest of the M1 network. Similar to cathodal tDCS-induced effects, the significant decrease in the characteristic path length induced by anodal tDCS was not accompanied by a significant increase in the connectivity degree. Therefore, the increase in efficiency does not depend on an increase in the number of functional connections, but is rather due to a reorganization of the functional network. Thus, our results provide for the first time evidence that the promotion of LTP-like plasticity induced by anodal tDCS [16] might be related to an efficient reorganization of the functional architecture of M1. Interestingly, a recent study shows that both anodal and cathodal tDCS over M1 induces a change in the generalization of the intrinsic coordinates of movement representations within M1 [50]. In that study the authors speculated that this change could result either from larger recruitment of the neurons during learning (increase in population number) or from a larger modification of the activity the respective neurons (increase in modulation), thus suggesting that the behaviourally quantified generalization patterns are due to tuning properties of neurons in specific networks within M1. Our results may in part confirm these hypotheses.

In a *post-hoc* analysis we investigated whether the tDCS-induced modifications of functional architecture may depend on the baseline functional organization of the identified clusters in Figure 3. We found that the effect of cathodal tDCS on the clustering coefficient strongly depends on the efficacy of the local connectedness before stimulation (Figure 4A). The same was true for the effects induced by anodal tDCS, where the baseline efficacy in the communication of the detected cluster in Figure 3C with the rest of M1 was positively correlated with anodal tDCS-induced alterations (Figure 4B). Interestingly, motor cortex plasticity induced by paired associative stimulation has been shown to be more effective in physically active than in sedentary individuals [51]. The investigators speculated that participation in regular physical activity may offer global benefits to motor cortex function that makes easier to enhance neuroplasticity. In principle accordance, the results of the present study suggest that the more efficient the functional architecture of M1 is at baseline, the more efficient the tDCS-induced functional modulations are. Following this concept, we hypothesized that the reason for the arm/hand region to be significantly altered by tDCS during rest is that this is the M1 region with the most efficient dynamic architecture. To test this hypothesis, we carried out a second *post-hoc* analysis, where we mapped the nodes that communicate more efficiently independently from stimulation (i.e. before tDCS intervention) within the M1 network. To this end, the average *L* maps for all subjects and all before-tDCS fMRI scans were averaged. Interestingly, we found that the regions where we found the significant tDCS-induced alterations belong to the largest M1 hub which is located at approximately the arm/hand region (Figure 5).

The substrate for plasticity induction within M1 is most likely a system of horizontal connections that spans M1, which may mediate the formation of associations among populations of M1 neurons and have been repeatedly shown in several studies to have the capacity for long-lasting synaptic modification [52], [53], [54]. Altogether, our results suggest that the mechanisms of the excitability shifts induced by tDCS (primary membrane hyper- and de-polarization by cathodal and anodal tDCS respectively, which results in NMDA receptor-dependent alterations of synaptic strength [12]) may take advantage of this M1 intrinsic circuitry, which supports the previously mentioned optimal conditions for network reorganization.

One of the most studied and well established outcomes of tDCS-induced neuroplasticity over M1 is the alteration of the size of motor evoked potentials (MEPs) as a measure of regional plasticity. Anodal tDCS increases and cathodal tDCS decreases the MEP size respectively, providing evidence for tDCS polarity-dependent neuroplasticity [10], [11]. The exact relation between this regional neuroplasticity and alterations of functional connectivity is so far unclear. Hereby, connectivity modulations induced by tDCS – as shown in the present study – might hint to a neuroplastic effect of tDCS on functional connectivity. However, this should be explored more directly in future studies. Additionally, it is important to notice that in the present study we evaluated the effects of tDCS alone – i.e. no motor training or learning was performed during or after the application of the stimulation. Thus it cannot be derived from the results of the study if tDCS improves motor learning due to its impact on functional connectivity. Since we have shown that tDCS reorganizes the functional architecture at the local level, and task-dependent alterations of functional connectivity have been demonstrated in other studies, this is however a tempting speculation which should be tested directly in future experiments.

Summarizing, in the present study we have shown that neuroplasticity induced by non-invasive stimulation over the primary motor cortex results in a reorganization of its functional architecture. This extents or knowledge about stimulation-induced alterations of brain functions relevantly beyond local excitability changes. The behavioural relevance of these alterations should be explored in forthcoming studies. We also show here that alterations of functional architecture can be imaged and mapped using graph theory at the voxel resolution level. Since the respective technique is suited to explore stimulation-induced alterations of functional connectivity throughout the brain, it might be an attractive tool to look for respective changes also in areas not easily addressed by surface EEG or excitability evoked potential measures alone.

## Supporting Information

Figure S1 **Shown is a 3D reconstruction of the T1 image of one of the subjects during a MRI scanning session (A).** The red rectangle shows the approximate location of the electrode over the scalp of the subject (A) and the surface average of all the subjects used in the present study (B). The left side of panel C shows the approximate boundaries of the tDCS electrode over the flattened representation of theleft BA4 (see figure 3A in the main text). On the right side of the panel C we show a rough approximation of the leg (purple), hand (green) and face (yellow) areas based on [43].
(TIF)

Figure S2 Shown are the left BA4 connectivity matrices for one of the subjects in all of the six resting state conditions (time*stimulation). The scale represents the Pearson's correlation value. The matrix in the before sham condition was thresholded and transformed to undirected graphs (Figures S3 and S4).
(TIF)

Figure S3 **The matrix in the before sham condition (upper left matrix in figure S2) was thresholded at T = 0.352 transformed to an undirected graphs.** Nodes were grouped in leg, arm and face subregions according to the division proposed in figure S1C. The values of the network parameters computed in the present study are shown in the upper left corner of the figure.
(TIF)

Figure S4 **The matrix in the before sham condition (upper left matrix in figure S2) was thresholded at T = 0.352 transformed to an undirected graphs.** Nodes were grouped in leg, arm and face subregions according to the division proposed in figure S1C. The graph is presented used a ring layout. The values of the network parameters computed in the present study are shown in the upper left corner of the figure.
(TIF)
