# Supplementary figures and images for: Reorganizing the Intrinsic Functional Architecture of the Human Primary Motor Cortex during Rest with Non-Invasive Cortical Stimulation
Source: PLoS One. 2012 Jan 27;7(1):e30971. doi: 10.1371/journal.pone.0030971 (PMC3267735; doi:10.1371/journal.pone.0030971)

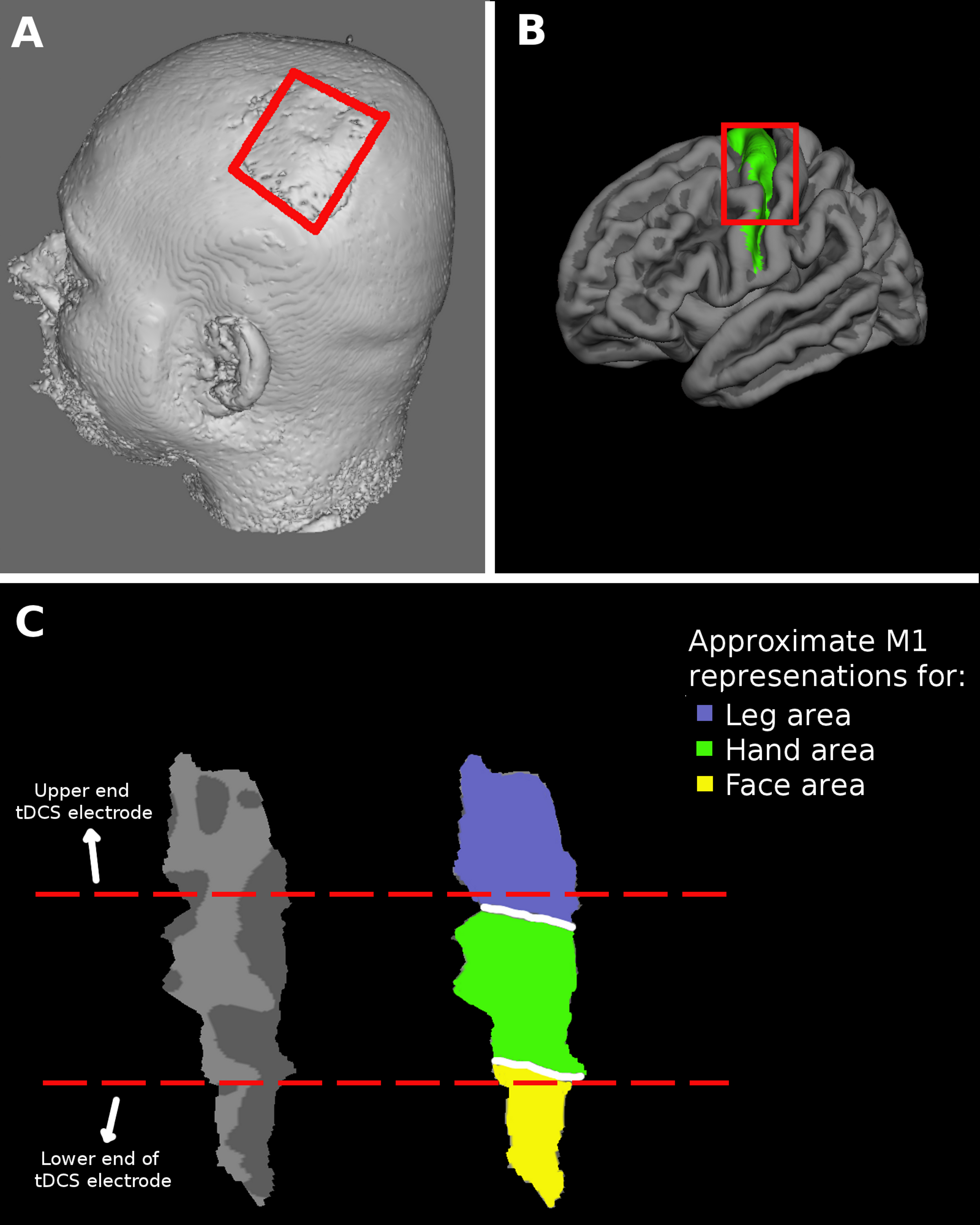

Supplement: Figure S1 — Shown is a 3D reconstruction of the T1 image of one of the subjects during a MRI scanning session (A). The red rectangle shows the approximate location of the electrode over the scalp of the subject (A) and the surface average of all the subjects used in the present study (B). The left side of panel C shows the approximate boundaries of the tDCS electrode over the flattened representation of theleft BA4 (see figure 3A in the main text). On the right side of the panel C we show a rough approximation of the leg (purple), hand (green) and face (yellow) areas based on [43]. (TIF) [file pone.0030971.s001.tif]

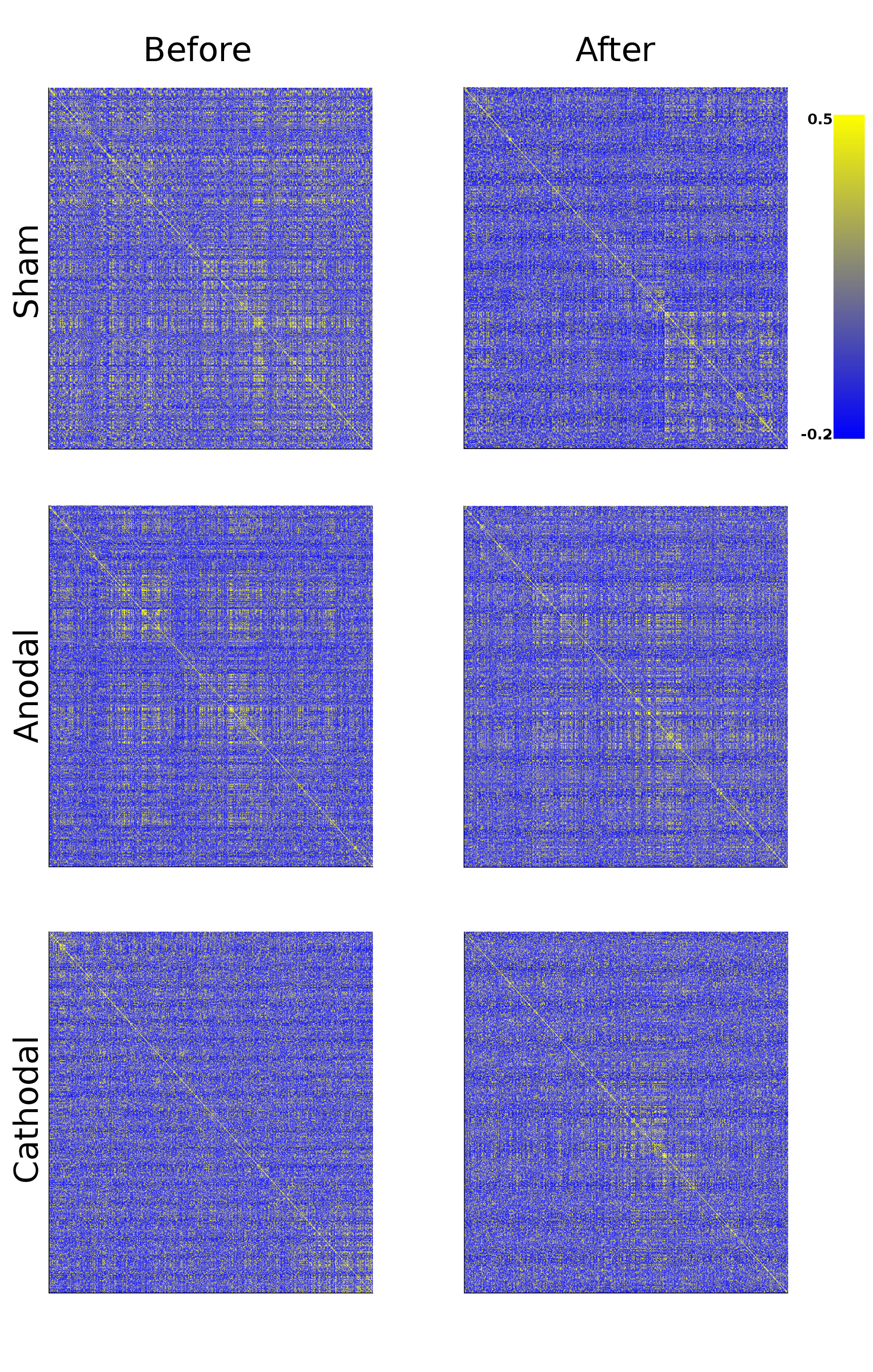

Supplement: Figure S2 — Shown are the left BA4 connectivity matrices for one of the subjects in all of the six resting state conditions (time*stimulation). The scale represents the Pearson's correlation value. The matrix in the before sham condition was thresholded and transformed to undirected graphs (Figures S3 and S4). (TIF) [file pone.0030971.s002.tif]

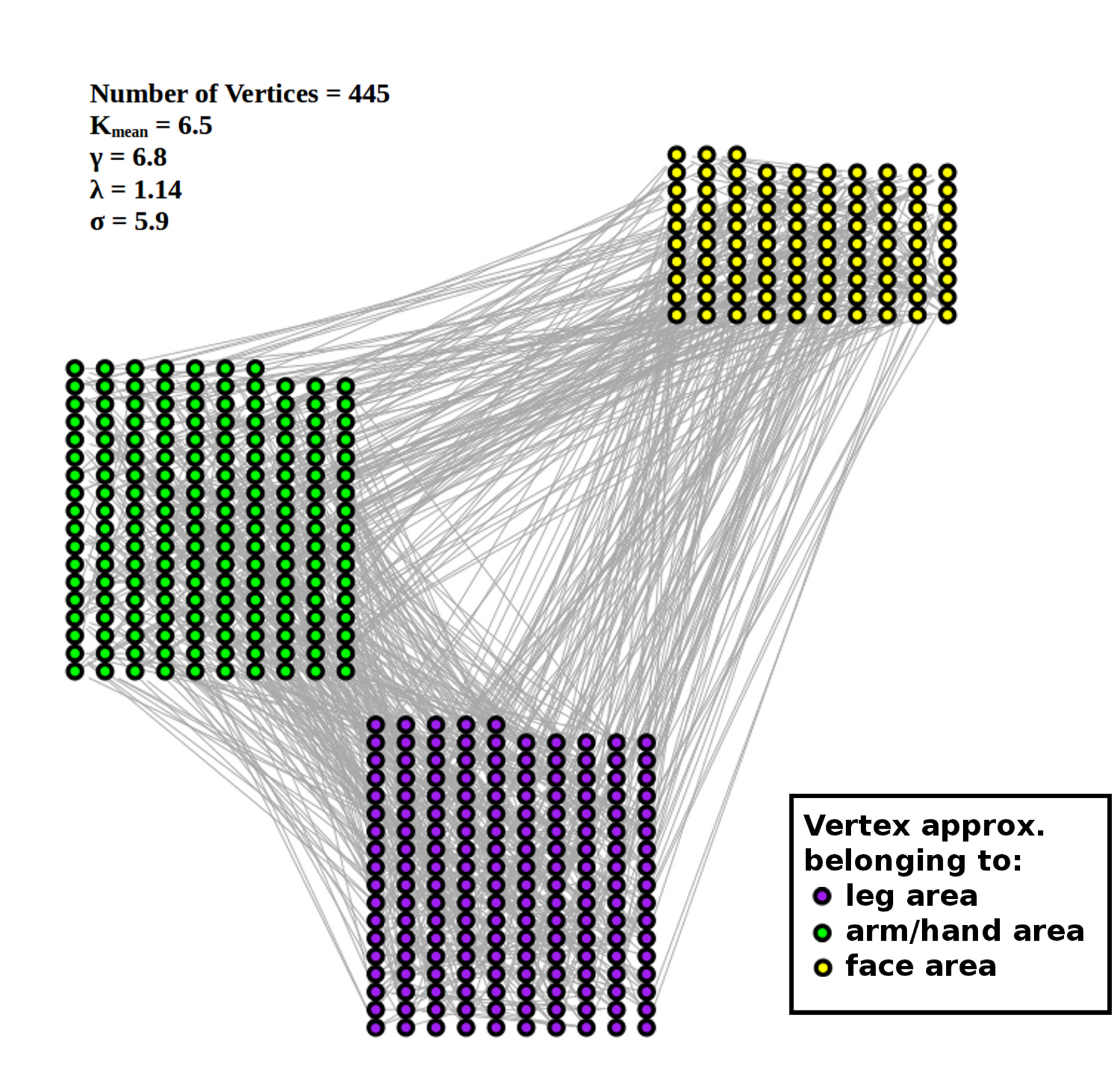

Supplement: Figure S3 — The matrix in the before sham condition (upper left matrix in figure S2) was thresholded at T = 0.352 transformed to an undirected graphs. Nodes were grouped in leg, arm and face subregions according to the division proposed in figure S1C. The values of the network parameters computed in the present study are shown in the upper left corner of the figure. (TIF) [file pone.0030971.s003.tif]

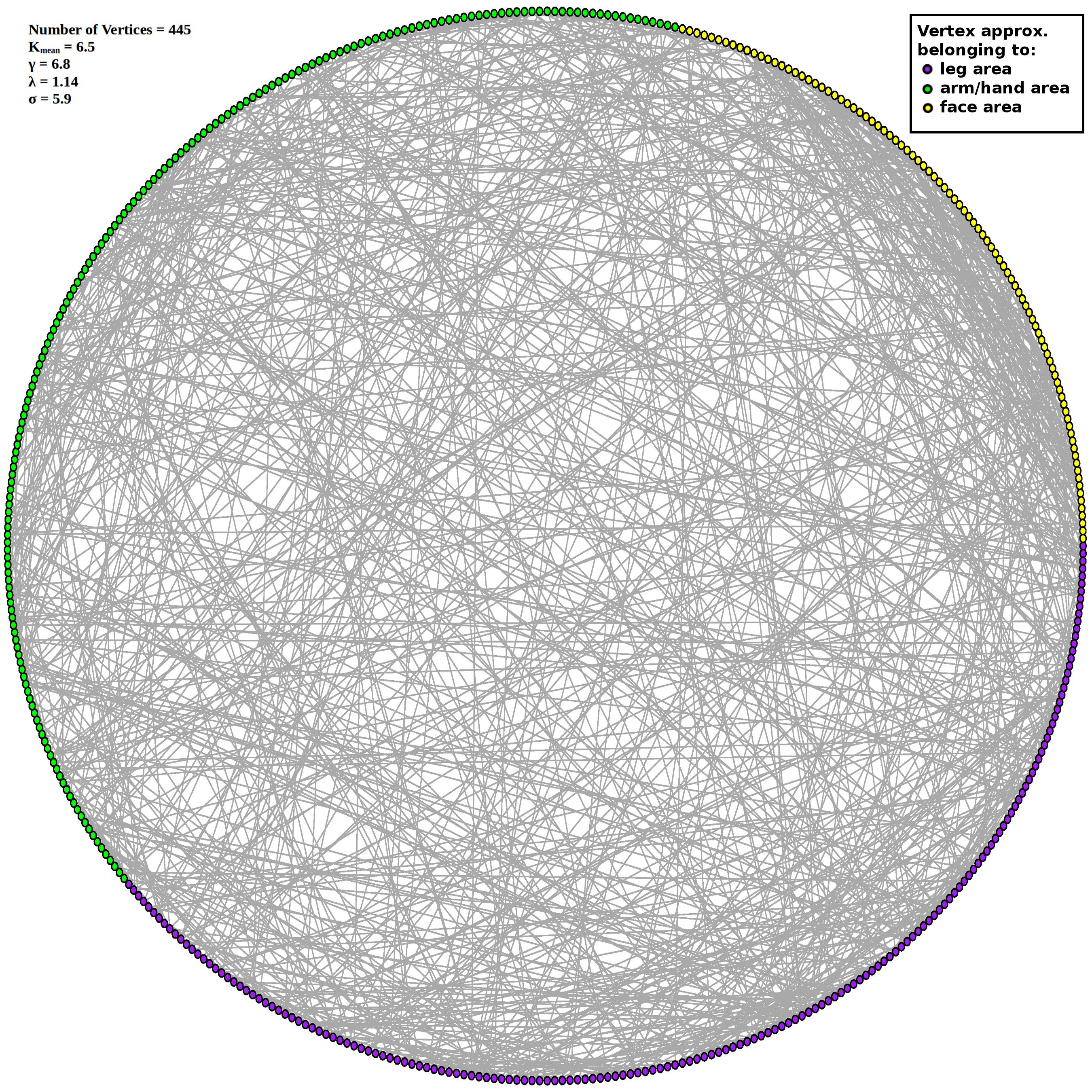

Supplement: Figure S4 — The matrix in the before sham condition (upper left matrix in figure S2) was thresholded at T = 0.352 transformed to an undirected graphs. Nodes were grouped in leg, arm and face subregions according to the division proposed in figure S1C. The graph is presented used a ring layout. The values of the network parameters computed in the present study are shown in the upper left corner of the figure. (TIF) [file pone.0030971.s004.tif]
